# Reporting practices for secondary findings among ERN GENTURIS member institutions in 15 European countries

**DOI:** 10.1038/s41431-026-02044-7

**Published:** 2026-03-03

**Authors:** Kathrin Taxer, Katharina Wimmer, Karin Wadt, Simon Schnaiter, Sabine Rudnik, Johannes Zschocke, ERN GENTURIS Study Group, Svetlana Bajalica-Lagercrantz, Irma van de Beek, Hilde Brems, Robin de Putter, Lenka Foretova, Maria K. Haanpää, Frederik J. Hes, Tiina Kahre, Barbara Klink, Mateja Krajc, Marina Macchiaiolo, Milan Macek, Arjen Mensenkamp, Antonio Percesepe, André Reis, Alessandra Renieri, Tim Ripperger, María Constanza Roa Bravo, Manuel R. Teixeira, Pavel Tesner, Philippe Theis, Thomas van Overeem Hansen, Fatima Vaz, Hildegunn Høberg Vetti, Gunda Schwaninger

**Affiliations:** 1 Institute of Human Genetics, Medical University Innsbruck, Innsbruck, Austria; 2 Department of Clinical Genetics, Rigshospitalet, Copenhagen University Hospital, Copenhagen, Denmark; 3 Department of Clinical Medicine, Faculty of Health and Medical Sciences, University of Copenhagen, Copenhagen, Denmark; 4 Department of Clinical Genetics, Cancer Genetic Unit, Karolinska University Hospital Solna, Stockholm, Sweden; 5 Department of Clinical Genetics, The Netherlands Cancer Institute, Amsterdam, The Netherlands; 6 Department of Human Genetics, University of Leuven, Leuven, Belgium; 7 Clinical Genetics Department, Ghent University Hospital, Ghent, Belgium; 8 Masaryk Memorial Cancer Institute, Brno, Czech Republic; 9 Department of Genomics, Turku University Hospital, Turku, Finland; 10 Clinical Sciences, Research Group Reproduction and Genetics, Centre for Medical Genetics, Vrije Universiteit Brussel (VUB), Universitair Ziekenhuis Brussel (UZ Brussel), Brussels, Belgium; 11 Department of Genetics and Personalized Medicine, Institute of Clinical Medicine, University of Tartu, Tartu, Estonia; 12 Department of Laboratory Genetics, Genetics and Personalized Medicine Clinic, Tartu University Hospital, Tartu, Estonia; 13 Medical Genetics Center (MGZ), Munich, Germany; 14 Department of Clinical Cancer Genetics, Institute of Oncology Ljubljana, Ljubljana, Slovenia; 15 Rare Diseases and Medical Genetics Unit Bambino Gesù Children’s Hospital Rome, Rome, Italy; 16 Department of Biology and Medical Genetics, Second Faculty of Medicine, Motol University Hospital, Charles University, Prague, Czech Republic; 17 Department of Human Genetics, Radboud University Medical Center, Nijmegen, the Netherlands; 18 Medical Genetics, University Hospital of Parma, 43126 Parma, Italy; 19 Institute of Human Genetics, Universtitätsklinikum Erlangen, Friedrich-Alexander-Universität Erlangen-Nürnberg, Erlangen, Germany; 20 Medical Genetics, University of Siena, Siena, Italy; 21 Department of Human Genetics, Hannover Medical School, Hannover, Germany; 22 Institute for Clinical Genetics, University Hospital Carl Gustav Carus at the Technische Universität Dresden, Dresden, Germany; 23 Department of Laboratory Genetics, Portuguese Oncology Institute of Porto (IPO Porto) / Porto Comprehensive Cancer Center, Porto, Portugal; 24 Cancer Genetics Group, IPO Porto Research Center (CI-IPOP) / RISE@CI-IPOP (Health Research Network), Portuguese Oncology Institute of Porto (IPO Porto) / Porto Comprehensive Cancer Center, Porto, Portugal; 25 Laboratoire national de santé (LNS), National Center of Genetics (NCG), Dudelange, Luxembourg; 26 Department of Clinical Genetics, Copenhagen University Hospital, Copenhagen, Denmark; 27 Instituto Português Oncologia de Lisboa Francisco Gentil, Lisbon, Portugal; 28 Western Norway Familial Cancer Center, Haukeland University Hospital, Bergen, Norway

**Keywords:** Genetic testing, Social sciences

## Abstract

Secondary findings (SF) identified in massive parallel sequencing raise important clinical and healthcare related questions. To get an overview on current practices of European healthcare providers (HCP), we conducted a cross-sectional survey study among 39 stakeholders—predominantly senior medical and laboratory geneticists—from 15 European countries participating in the European Reference Network for Genetic Tumour Risk Syndromes (ERN GENTURIS). Respondents reported considerable heterogeneity in SF management and reporting, even within countries. While 31% of responding HCP return findings from all 81 genes on the American College of Medical Genetics and Genomics (ACMG) recommended SF list version 3.2, 41% restrict SF disclosure, often excluding genes associated with cardiological or metabolic disorders or with limited clinical actionability. A further 26% do not report ACMG-listed SF at all. Notably, 70% of HCP also assess additional cancer-predisposition genes beyond the ACMG list, using in-house gene lists or national guidelines. Most HCP restrict reporting to (likely) pathogenic variants (90%) and find SF in less than 5% of genetic analyses (59%). Consent procedures and patient information practices varied, with most HCP employing opt-in consent models and genetic counselling primarily delivered by medical geneticists and genetic counsellors. Major institutional challenges raised by participants, include lack of harmonised guidelines, concerns about patient anxiety, and insufficient resources for follow-up care. The findings of this study highlight the need for robust, evidence-based European guidelines to ensure clinically relevant and patient-centred SF management.

## Introduction

The European Reference Network for Genetic Tumour Risk Syndromes (ERN GENTURIS) is a collaborative initiative established in 2017 as one of the 24 European Reference Networks [1]. Spanning 23 European countries, ERN GENTURIS unites expert healthcare providers (HCP) to enhance diagnosis, treatment, and care of individuals affected by genetic tumour risk syndromes [2].

With the emergence of massively parallel sequencing, it has become increasingly feasible to actively and systematically identify secondary findings (SF). SF are defined as genetic variants that are unrelated to the primary reason for genetic testing and are actively searched for during genetic testing, a practice often referred to as opportunistic screening. In contrast to incidental findings, which are also unrelated to the primary indication but are unintentionally discovered during genetic analyses. The practice of opportunistic screening for SF remains a topic of ongoing debate regarding its clinical utility, ethical implications [3], and practical implementation [4, 5]. Proponents argue that reporting SF may enable disease prevention or early diagnosis, particularly for conditions with established surveillance or intervention strategies [6, 7]. However, concerns persist regarding resource allocation, informed consent, legal issues, equity of access, and the management of surveillance and interventions, especially in asymptomatic individuals from unaffected families [8, 9]. The frequency of SF identified through exome and genome sequencing is estimated at 1–6% in large population studies [10–15] depending on the gene list used, interpretation and inclusion of variants and the study population [16]. Pathogenic and likely pathogenic variants in genetic tumour risk syndrome-related genes (cancer-related genes, CRG) are present in approximately 1–2% of individuals of Central European descent [10, 17]. The majority of SF are found in genes associated with Hereditary Breast and Ovarian Cancer (HBOC) and Lynch Syndrome, underscoring the potential impact of SF reporting on patient surveillance and management [17]. The interpretation and classification of SF are further influenced by differences in gene panel design and the definition of narrow vs. broad clinical indications across institutions. Multigene panel testing—in which several genes associated with a specific clinical condition are analysed simultaneously, is commonly used in cancer genetics. Depending on the definition of the primary indication (e.g. HBOC vs. cancer predisposition), panels are defined inconsistently and must be clearly distinguished from opportunistic screening [4].

This study aims to elucidate the current practices for the reporting of SF among ERN GENTURIS HCP, to identify challenges in implementation, and to inform the development of ethically and practically sound guidelines for the management of SF in genetic testing.

## Current reporting practices for secondary findings

International guidelines for the reporting of SF remain heterogeneous. The American College of Medical Genetics and Genomics (ACMG) recommends the return of (likely) pathogenic variants in a curated list of 81 genes as of version 3.2, including 28 CRG, regardless of the original testing indication [6, 18]. The ACMG guidelines are constantly updated and refined and are widely referenced but not universally adopted in Europe, where country-specific policies are limiting and often inconsistent [18–21]. Exceptions are organisations, such as the French Society of Predictive and Personalised Medicine (SFMPP) and Genomics England, who also support the return of genetic test results retrieved from opportunistic screening and have developed their own gene lists and classification systems [7, 22]. The SFMPP, for example, categorises genes into three classes based on risk and actionability, with only the most actionable genes recommended for routine reporting [7]. Both the ACMG and SFMPP advise against reporting findings with limited clinical utility or technical complexity, such as those involving mitochondrial DNA or pharmaco-genomic variants [6, 7]. Other institutions such as the European Society of Human Genetics (ESHG) and the Canadian College of Medical Genetics recommend a more cautious approach, opposing the routine search for SF. They recommend that genetic testing remains as targeted as possible with bioinformatics filtering for genome-wide analysis or extended gene panels covering several indications [4, 23]. While the ESHG advocates for an opt-in informed consent process in cases where SF are investigated and restricts the reporting of SF in minors to childhood-onset conditions [4, 13, 24], the ACMG recommends an opt-out consent model and supports the disclosure of adult-onset conditions in children when it is assumed to be in the minor’s ‘best long-term interest’ [6]. The use of dynamic consent, supporting ongoing communication and allowing for changing preferences of patients, was found to be preferred by stakeholders providing exome and genome sequencing for the investigation of SF [25] and is also proposed by the SFMPP [7].

## Subjects and methods

### Survey development and distribution

**Fig. 1:**
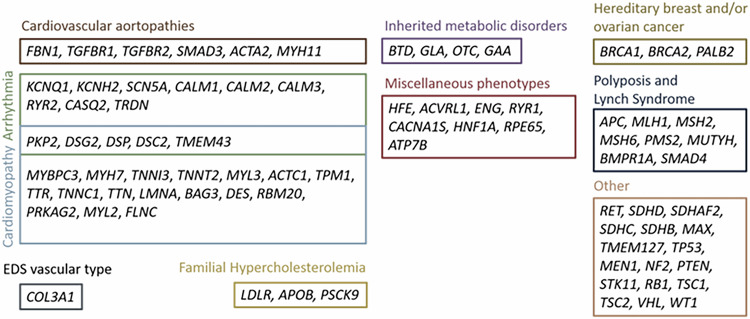
A grouped version of the 81 genes included in ACMG secondary findings list version 3.2. This figure was provided to participants during the survey when questions were relating to the ACMG gene list to inform participants about the different phenotype categories in ACMG SF v3.2. There are 28 cancer-related genes shown on the right side of the figure.

An online questionnaire was developed using Lime Survey (Medical University of Innsbruck license), a secure, web-based survey platform. The questionnaire underwent extensive internal and external review within ERN GENTURIS representatives to ensure content validity and clarity. All survey items were presented in English, considering the high educational baseline of participants, and pre-tested for readability and comprehension. The survey comprised up to 34 items, including single-choice, multiple-choice, and open-ended questions, depending on branching logic (survey with all questions, see Supplementary 1). Items covered professional demographics, institutional practices, and attitudes towards reporting SF. The ACMG SF list v3.2, with 81 genes including 28 CRG, was used provided in form of a grouped figure in the survey (Fig. 1) [18]. An open-text field for additional comments was provided in many questions and at the end of the survey. The survey was distributed via email to all members of the ERN GENTURIS, encompassing 180 individuals from 51 HCP across 23 European countries (22 EU countries and Norway). Participation was voluntary and anonymous, with the option for respondents to provide their names and/or institutions for acknowledgement in the publication. The survey was open from March 13 to April 12, 2025, and two reminder emails were sent to maximise the response rate.

### Data management and analysis

Survey responses were exported from Lime Survey into Microsoft Excel for data cleaning and analysis. Duplicate entries and responses missing the minimal dataset were excluded to ensure data integrity; in two cases of duplicate submissions from the same person, only the more recent entry was retained. In cases where the number of responses was higher than the number of national HCP all responses were retained, treated, and analysed individually, analogous to responses originating from separate centres, even if there was a discordance between answers. This was done because the analytical approach did not allow to determine which response should be considered more valid or representative. Descriptive statistics were used to summarize demographic characteristics and distribution of responses to key survey items. Percentages reported, reflect valid responses, excluding missing data, as participants could skip some questions while most required a response. For open-ended responses, content analysis was conducted as a systematic qualitative research method to interpret and categorise free text data. By coding free text survey responses recurring themes and underlying factors were identified to provide deeper insights into participants’ attitudes and practices. All data handling procedures adhered to ethical standards for research involving human participants, with strict attention to confidentiality and accurate reporting of findings. The full data analysis was performed by two researchers.

## Results

### Survey response and participant characteristics

A total of 56 individuals responded to the survey. After excluding 17 incomplete or duplicate responses, 39 complete and valid responses were included in the analysis (22% individual response rate). On the institutional level, participants represented at least 58% (30 out of 51) of ERN GENTURIS HCP. This is a minimum number, since respondents could complete the survey anonymously and therefore without specifying their HCP, not all responses could be directly linked to a specific HCP.

The survey achieved broad geographic representation, with responses from 15 European countries, covering 65% (15 out of 23) of ERN GENTURIS member countries. The highest number of respondents was received from Germany (*n* = 9; representing at least 4 of 6 German HCP), followed by Italy and the Czech Republic with five respondents each (covering at least 5 of 9 HCP in Italy and both HCP in the Czech Republic).

**Fig. 2:**
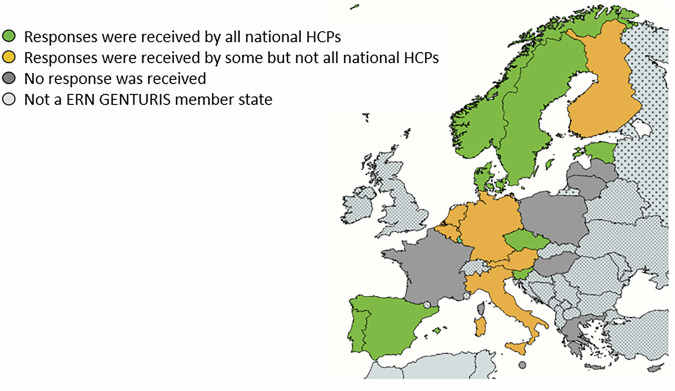
Response map of participating healthcare providers. There are a total of 23 ERN GENTURIS member states (shown in solid colours green, orange, and grey). Coloured in green are countries from which all ERN GENTURIS healthcare providers responded to the survey. From countries, coloured in orange participants from a part of the healthcare providers responded to the survey. From countries coloured in grey no responses were received. Countries shown in grey dots are not member states of ERN GENTURIS. (Source: World Map - Simple | MapChart).

**Fig. 3:**
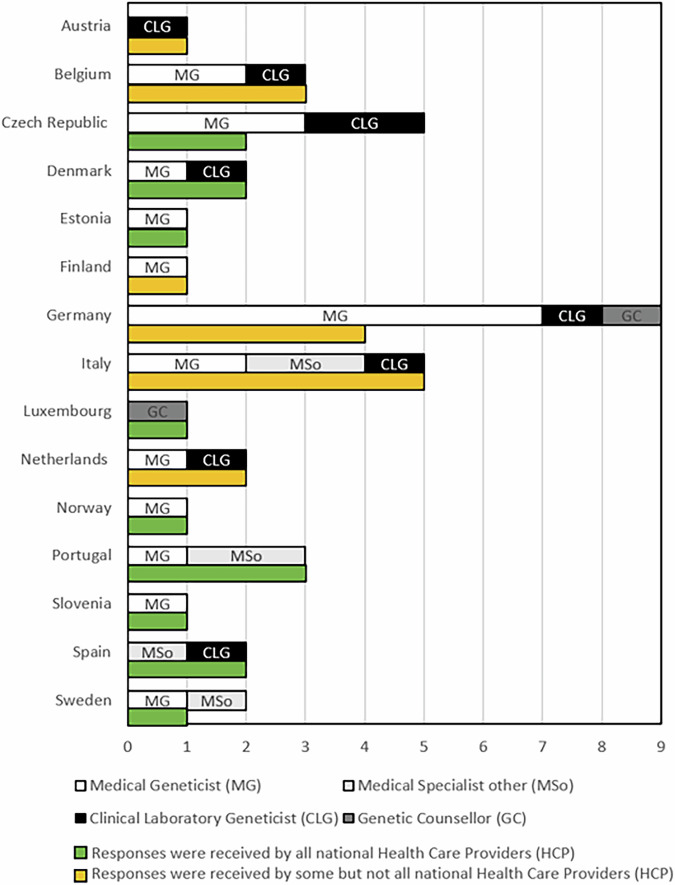
Countries, professions and distribution of participants. Participating countries are listed on the y-axis, with the total number of responses per country shown on the x-axis. For each country, professions of the respondents are displayed in the upper bar, using white, black, and two intensities of grey to represent Medical Geneticists (MG), Clinical Laboratory Geneticists (CLG), Other Medical Specialists (MSo), and Genetic Counsellors (GC). The abbreviation of each profession is stated in the bar. The lower bar indicates the total number of healthcare providers responding from each country. Green highlights countries where all national ERN GENTURIS HCP are covered, while orange indicates partial coverage (i.e., some but not all national HCP are represented).

Other participating countries included Austria (*n* = 1; 1 of 2 HCP), Belgium (*n* = 3; 3 of 3 HCP), Denmark (*n* = 2; 2 of 2 HCP), Estonia (*n* = 1; 1 of 1 HCP), Finland (*n* = 1; 1 of 2 HCP), Luxembourg (*n* = 1; 1 of 1 HCP), the Netherlands (*n* = 2; 2 of 5 HCP), Norway (*n* = 1; 1 of 1 HCP), Portugal (*n* = 3; 3 of 3 HCP), Slovenia (*n* = 1; 1 of 1 HCP), Spain (*n* = 2; 2 of 2 HCP), and Sweden (*n* = 2; 1 of 1 HCP). Most respondents represented a single HCP, although in Germany, the Czech Republic, and Sweden, multiple participants responded for the same institution (see Figs. 2 and 3). No responses were received from the following ERN GENTURIS member countries: Cyprus, France, Greece, Hungary, Lithuania, Latvia, Malta, and Poland. Among the 39 respondents, the majority identified as medical geneticists (*n* = 22), followed by clinical laboratory geneticists (*n* = 9), medical specialists from other disciplines (*n* = 6), and genetic counsellors (*n* = 2) (Fig. 3). Most participants (*n* = 34 of 39) had more than five years of professional experience, with 15 serving as heads of department and 6 as heads of laboratory units.

### Reporting Practices for Secondary Findings

**Fig. 4:**
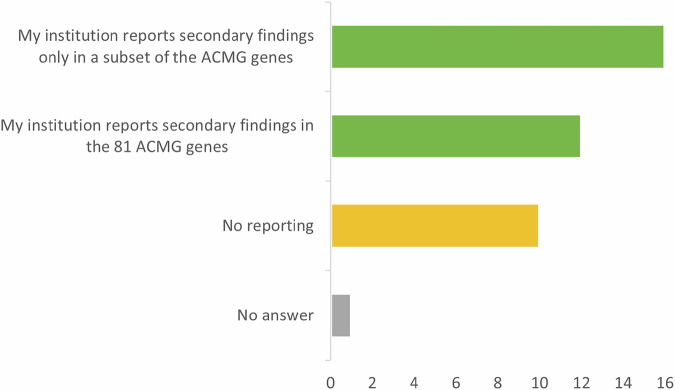
Reporting of secondary findings in the 81 ACMG genes varied among participants. Of 39 respondents, 28 (72%) reported secondary findings in at least some ACMG genes: Twelve (31%) reported secondary findings in all 81 genes, while 16 (41%) reported only a subset. Ten participants (26%) stated their institutions do not report secondary findings, and one (2%) did not answer.

The HCP reporting SF can be split into two groups, one reporting SF in only a subset of ACMG genes (*n* = 16 from at least 13 HCP) and the other reporting them in all 81 ACMG genes (*n* = 12 from at least 8 HCP). Participants in the first subgroup were asked for their reporting practice. When analysing the 15 free text answers received, using content analysis, three major themes appeared whereby not every theme was always named individually but sometimes two of the themes were named together: (1) The focus of the HCP is mainly on cancer predisposition syndromes reported for five HCP, (2) the use of cancer gene panels that do not include all ACMG genes which was reported seven times, and (3) the absence of specific guidelines for surveillance for the full list of ACMG conditions which was mentioned six times. Furthermore, among those 16 participants that said their HCP report a subset, 11 participants (69%) stated to exclude cardio-related genes and 10 (63%) stated to exclude metabolic-related genes. Here, one person specifically answered to not report SF in the genes *COL3A1, BTD, GLA, OTC*, and *GAA* in free text while other participants would not report SF in specific CRG (genes named once: *BMPR1A*, *SMAD4*, *SDH*x, *RB1*; genes named twice: *WT1*, *TSC2*, *TSC1*, *NF2*, *TMEM127*, *MAX*). An additional participant specifically mentioned not to report variants in *PMS2*. Ten participants (26%) said that their institutions do not report SF at all, and one (2%) did not provide an answer (Fig. 4).

### Reporting of cancer-related genes (CRG)

Thirty participants answered the question regarding the reporting of SF in additional CRG. While four participants (13%) adhered to the reporting of SF in the 28 CRG of the ACMG list in their HCP, and five participants (17%) did not know, 21 participants (70%) stated that their HCP reports additional CRG not included on the ACMG list. Of these 21, 16 (76%) used HCP-specific in-house gene lists, and five (24%) relied on international guidelines, such as the UK National Comprehensive Cancer Network Guidelines referenced by participants. The number of additional genes in SF reporting ranged from a single gene (e.g., *FLCN*) to as many as 260 genes (for all provided gene lists, see Supplementary 2). According to content analysis of free text responses, reported genes were most commonly selected based on medical actionability, available screening, research focus, and carrier frequency.

### Frequency of secondary findings and reported variant classes

Twenty-two participants answered the question regarding frequency and variant classes. The frequency of SF detected in genetic analyses varied considerably. Three participants (14%) stated that SF are found in more than 5% of genetic analyses, thirteen participants (59%) reported to find SF in 1–5%, and five participants (23%) in less than 1% of genetic analyses. One participant (4%) reported SF in another frequency, not further specified. Of the 22 participants that answered the question about variant classes, 20 (90%) reported only (likely) pathogenic variants (ACMG classes 4 and 5 [26]), while two (10%) also reported variants of uncertain significance (ACMG class 3 [26]).

### Reporting practices in trio analysis

Thirty participants gave insight in their practice of performing (postnatal) trio exome or genome sequencing. Twelve participants (40%) stated to report SF in the 81 ACMG genes, seven (23%) stated to not report SF in trio analyses at all, and eleven (37%) were unsure. Among those reporting SF in trios, five (42%) reported SF in all three individuals in the trio, while seven (58%) first analysed the index case and only extended analysis to relatives if a variant was found in the index.

In the context of CRG, exome or genome sequencing was performed by only six participants (15%), while most, 32 participants (82%), used panel analysis for cancer-related genetic testing, one (3%) did not provide an answer to this question.

### Rationale for broader reporting of secondary findings in cancer panels

When asking in more detail about the practice of reporting SF in cancer panels, the 24 participants (62%) who stated that SF were identified within panel analysis were asked about their reasoning. The content analysis revealed that cancer genetics laboratories across institutions employ broad, often customised panels and screen for several (overlapping) cancer predisposition syndromes. Especially the HBOC and Lynch Syndrome genes were mentioned multiple times to be investigated in patients with either HBOC or Lynch Syndrome as well as other cancers. It was further stated, that in the setting of predictive testing for a known familial mutation in healthy individuals, other genes are investigated in addition to the familial variant. The same approach of broader testing was reported to be offered to patients that are tested for the eligibility of PARP inhibitor treatment. Participant quotes provide insights on the practices for reporting SF in panel analysis in the context of cancer-related genetic testing (Table 1).

**Table 1:** Representative quotes illustrating institutional practices in cancer predisposition genetic screening using broad panel testing across cancer types.

Clinically overlapping cancer predisposition syndromes, e.g. MLH1, MSH2 and MSH6 is looked for in all patients with suspected HBOC screening, not PMS2.
All probands predictively tested for familial HBOC-associated variants are screened for other (l)pV in all 13 HBOC-associated genes, plus MLH1, MSH2, MSH6 - not PMS2.
All probands tested for PARPi indication (i.e, BRCA1, BRCA2) are screened for other (l)pV in all 13 HBOC-associated genes plus MLH1, MSH2, MSH6 - not PMS2.
A customized NGS cancer panel that includes all known tumour predisposition genes is used uniformly for all cancer patients, to also screen for other cancer predisposition genes that may not be directly related to the initial suspicion.
We test with a broad cancer gene panel, and this means we also test breast cancer genes in CRC patients and vice versa.
We use a whole multigene panel for hereditary cancer (260 genes) in any patient tested for hereditary cancer predisposition.
We use an extensive cancer predisposition gene panel, and report for all the indications SF according to the ACMG. We do not want to risk that other malignancies will develop in the future without reporting.

*HBOC* Hereditary Breast and Ovarian Cancer, *(l)pV* (likely) pathogenic variant, *MMR* Mismatch repair, *NGS* Next-generation sequencing; *PARPi* Poly (ADP-ribose) polymerase inhibitor, *CRC* Colorectal cancer, *ACMG* American College of Medical Genetics and Genomics, *SF* Secondary findings.

### Patient counselling and consent practices

Twenty-one participants specified how information regarding SF is provided to patients. Ten participants (48%) defined pre-test information regarding SF as general and eleven participants (52%) as more specific. Sixteen of the 21 participants (76%) that provided information on the consent process, confirmed that an opt-in consent model for SF reporting was used at their HCP, three (14%) used an opt-out consent model, and two (10%) were unsure. Fifteen participants specified by whom pre-test counselling was given. Ten participants (67%) said that it was provided by medical geneticists, four (27%) referred to genetic counsellors and one (6%) mentioned other medical specialists. Post-test disclosure of SF was described in more depth by 28 participants. It was said to be delivered by medical geneticists stated by 21 (75%), followed by genetic counsellors stated by five (18%), and other medical specialists stated by two (7%). Ninety percent of participants provided the information on the SF in the same appointment as the primary results, while 10% offered to discuss the SF in a separate appointment. One of these participants noted that SF are discussed during the same appointment and delivered in one laboratory report when the patient has cancer and the SF is also related to hereditary cancer risk.

### Concerns regarding reporting practices

Among the 28 responders from HCP reporting SF, the most frequently cited concerns were the lack of guidelines (*n* = 11), potential patient anxiety (*n* = 9), and the reporting of adult-onset disease in children (*n* = 7). Other concerns included insufficient surveillance programs, limited counselling capacity, and cost of insurance coverage for surveillance. Responders from institutions not reporting SF (*n* = 10) were asked about their reasoning. This group had less and different concerns. Main reasons for not reporting SF were insufficient resources (*n* = 3), risk of genetic discrimination (*n* = 2), and issues related to insurance, reporting in children, patient anxiety, and lack of guidelines (*n* = 1 each) (Table 2).

**Table 2:** Concerns on reporting in institutions that return secondary findings (*n* = 28) vs. do not return secondary findings (*n* = 10).

Concerns	Reporting SF (*n* = 28)	Not Reporting SF (*n* = 10)
• No concerns	6 (22%)	4 (40%)
• Risk for genetic discrimination		2 (20%)
• Insurance coverage for SF		1 (10%)
• Insurance coverage for surveillance	1 (4%)	
• Lack of surveillance programs	6 (22%)	
• Reporting adult onset diseases in children	7 (25%)	1 (10%)
• Anxiety in patients	9 (32%)	1 (10%)
• Lack of counselling capacity	4 (15%)	1 (10%)
• Insufficient resources (time, finances, personnel)		3 (30%)
• Missing guidelines	11 (40%)	1 (10%)

The same set of concerns was presented to both groups as a multi-answer item with free text option. The two columns show the absolute numbers of participants that chose each concern on the list. Multiple answers were possible. Abbreviation: SF: Secondary findings.

## Discussion

This study provides an overview of current practices in reporting SF among ERN GENTURIS member institutions across 15 European countries. Our findings highlight considerable heterogeneity in institutional policies, gene lists, reporting strategies, and concerns. This reflects the ongoing international debate and lack of harmonised guidelines to screen and manage SF in Europe.

The survey revealed that a high proportion of 28 of 39 participants (72%) from ERN GENTURIS HCP, are reporting SF in part of or in all ACMG v3.2 list genes. Notably, many HCP in this study exclude entire gene categories, such as cardio- or metabolic-related genes, or specific genes with limited actionability or unclear clinical benefit for example SF in the *PMS2*-gene. In the setting of cancer-related testing, we observe, that practices for reporting are highly variable among HCP. In cancer patients, 21 of 30 participants (70%) analyse additional cancer-related genes while only four (13%) adhere to the ACMG cancer gene recommendations. As there are no national or international guidelines covering all additional cancer-related genes, most healthcare professionals instead apply HCP-specific in-house gene lists. However, some countries, for example Belgium, and the Czech Republic, appear to undertake efforts toward greater national standardisation by incorporating established international frameworks, like the ACMG or the UK National Comprehensive Cancer Network guidelines, which are used considering local healthcare capacity and reimbursement structures. Responses from Belgium showed that they are actively seeking consensus around the adoption of ACMG guidelines, signalling a broader interest in harmonising testing and reporting practices. The absence of responses from several ERN GENTURIS member countries restricts the breadth of these national perspectives captured. This is particularly relevant for countries such as France, where national recommendations exist for reporting SF. The lack of input from these countries may therefore underrepresent existing national frameworks from countries where structured approaches to SF are already in place, meaning that reporting of SF would likely have been even more prominent had countries such as France been represented. In other countries, like the Netherlands, national guidelines restrict genetic testing to the primary indication (personal correspondence). In many answers, the precise differentiation of panel analysis for specific indications and opportunistic screening was vague, probably due to diverging definitions of narrow vs. broad indications. For instance, most participants - 32 out of 39 (82%) - stated that their HCP uses cancer gene panel analysis for cancer patients and three quarters of them do not limit their analysis to genes associated with the primary indication, but rather analyse additional, if not all, genes present on their (custom) panels (Table 1). The provided rationale for this broad screening approach in cancer panel analysis was mainly stated to be the enhancement of surveillance, prevention, and patient care by identifying additional cancer risk variants. This shows, that although SF are conceptually defined as genetic findings unrelated to the primary clinical indication for testing, this distinction can become blurred in oncogenetics when multigene panel testing is used. Testing practices - such as analysis of extensive cancer gene panels, predictive testing of additional susceptibility genes, or testing to inform PARP inhibitor eligibility - may be interpreted either as the intentional broadening of the primary test scope or as the generation of SF, depending on testing intent, consent framing, and institutional norms. This dual interpretability has important implications for comparability across institutions. Differences in how SF are defined and operationalized may lead to variability in SF management, even when underlying testing practices are similar. Our findings therefore reflect not only variation in clinical practice but also heterogeneity in conceptualisation, underscoring the need for clearer and more harmonised frameworks to support consistent interpretation and reporting of SF across settings, also in multigene panel testing.

The widespread use of specific CRG lists in the ERN GENTURIS HCP reflects their specialisation on oncogenetics. According to content analysis this was mainly influenced by local expertise, specific genetic background, research focus, and available resources. In 72% of HCP reporting of SF in CRG was stated to go beyond the 28 CRG in the ACMG list. Our results show that many HCP prioritise genes with established medical actionability and available preventive measures, reflecting an approach to maximise patient benefit while minimising potential harm [9]. Large variation in the number of genes included in these lists further illustrates the lack of consensus and the need for robust, evidence-based guidance, on actionability of (likely) pathogenic variants both found in patients with a specific cancer predisposition syndrome as well as in asymptomatic individuals from unaffected families. For the purpose of this publication, we worked with version 3.2 of the ACMG SF list [18] since it was the most current version in March 2025 (Fig. 1). However, the ACMG recently updated their list to version 3.3, providing an online resource, taking into account which variants in a given gene should be reported [21].

In Europe and the United States, studies have demonstrated that the majority of patients, when given the choice, express a strong preference to be informed about SF [13, 17, 27]. Despite this clear patient interest, there remains a paucity of robust studies on the clinical, behavioural, and psychological impact of receiving SF [28, 29]. So far, available data provides little evidence that SF recipients suffer significant adverse effects [14] but more research is needed to determine if receiving SF can lead to effects like anxiety or decisional conflicts, particularly when the clinical significance of the finding is unclear or preventive options are limited [9, 29–31]. In recognition of these complexities, professional societies across Europe and the United States consistently emphasise the importance of offering opportunistic screening only within a framework of step-wise genetic counselling. Importantly, our survey results provide empirical support for these recommendations, demonstrating that experienced genetic professionals—namely medical geneticists and genetic counsellors—are routinely involved in delivering information and counselling [8, 32]. Moreover, our findings show that the recommended practice of restricting reporting to (likely) pathogenic variants is standard, thereby minimising patient insecurity related to variants of uncertain significance.

Despite the growing interest in SF reporting, our findings reveal persistent concerns among participating professionals, including the lack of clear guidelines, the potential for patient anxiety, limited resources for counselling and surveillance, and ethical concerns such as reporting adult-onset conditions in children. The observed differences in reporting practices both within and between countries likely reflect the diverse healthcare systems, the specialisation of participants in oncogenetics, regulatory environments, and ethical frameworks across Europe. The lack of harmonisation may also be influenced by differences in the interpretation of medical actionability, the availability of surveillance programs, and national reimbursement policies. These findings underscore the need for a coordinated European approach to SF reporting, with standardised criteria for gene and variant selection, clear consent processes, and robust support for both patients and healthcare professionals.

### Limitations

This study is subject to several limitations. The response rate, while high for the different countries and member institutions, was modest on the individual level. Furthermore, as the survey relied on self-reported data, responses may not always correspond to actual HCP practice. However, the observed consistency in responses from the same HCP suggests that a single response per institution, particularly as most responses come from department and laboratory heads, may provide a representative view of local practice. Despite this, the findings may not fully reflect the diversity of approaches across all ERN GENTURIS member institutions. Another influence might have been that some participating centres are predominantly specialised in cancer predisposition syndromes rather than a broader spectrum of genetic conditions. There is also a risk of self-selection bias, as individuals more actively involved in SF reporting may have been more likely to respond. This can lead to an overrepresentation of centres actively reporting SF and is potentially limiting the generalisability of the reported practices. Moreover, the survey was intended to better understand the reporting practices of SF within ERN GENTURIS members and therefore provides only limited information about the overall treatment of SF in rare disease diagnostics.

## Conclusions and future directions

In summary, our study demonstrates that the majority (72%) of participating ERN GENTURIS member institutions report SF to patients but there is considerable variability in this practice. Despite strong support for patient autonomy and the involvement of specialised genetics professionals in SF disclosure, significant challenges persist in developing guidelines, allocating resources for SF testing, genetic counselling, and clinical management. ERN GENTURIS working groups are well equipped to be part of the development of evidence-based European guidelines to help ensure equitable and patient-centred reporting of SF, particularly in oncogenetics, and to harmonise genomic medicine across Europe. Future research needs to focus on evaluating the effect of individual genetic variants on disease prognosis and outcomes of SF disclosure for patients and families, and development of support frameworks for the implementation of best practice across diverse healthcare systems.

## Supplementary information

Supplement 1

Supplement 2

## Data Availability

The datasets generated and analysed during the current study are available from the corresponding author upon reasonable request, subject to conditions ensuring participant confidentiality. Due to privacy considerations, raw data cannot be publicly deposited. Summary data supporting the findings of this study are included within the article and its supplementary materials.
