# Supplementary material for: Reporting practices for secondary findings among ERN GENTURIS member institutions in 15 European countries
Source: Eur J Hum Genet. 2026 Mar 3;34(8):1121–9. doi: 10.1038/s41431-026-02044-7 (PMC13424559; doi:10.1038/s41431-026-02044-7)
Supplement: Supplementary file 1 — Supplement 1 [file 41431_2026_2044_MOESM1_ESM.docx]

- 1. Please select the country you are working in.
  2. Which is the institution/laboratory you are working in?
  3. What is your profession?
  4. What is your current position?
  5. Would you like to be acknowledged as collaborator of the study in a "study group" - then please provide your name and contact details here. If you do not provide any name/contact details here your answers will be saved anonymous.

1. Does your institution actively look for and report secondary findings? YES means your institution always OR in specific cases looks for and reports secondary findings in various settings NO means your institution never looks for and reports secondary findings (If NO: What are your institute's primary concerns, because of which you are not reporting secondary findings? Do you think that in the near future secondary findings will be reported?)
2. In which genetic analyses are secondary findings routinely looked for and reported by your institution?
   1. Your institution reports secondary findings in panel analysis. Please provide a short explanation how and why secondary findings are investigated in panel analysis. (e.g. breast cancer genes are looked for in patients with suspected Lynch-Syndrome)
3. How does your institution generally analyse patients with a cancer predisposition syndrome (CPS)?
4. Your institution generally looks for and reports secondary findings. The ACMG defines 81 medically actionable genes and recommends secondary findings in these genes to be reported to patients unrelated to the indication in clinical genetic testing. (In genes associated with an autosomal-recessive inheritance, reporting is only recommended when 2 variants are detected.) Does your institution report secondary findings in the ACMG genes? (Miller DT et al., 2023)
   1. If you do report secondary findings in a subset of ACMG genes - in which genes do you not report them?
   2. If your institution reports secondary findings only in a subset of the ACMG genes, please provide a reasoning, why. (e.g. not all genes are included in the cancer panel used, or no guidelines for surveillance,...)
5. This questions only refers to cancer related genes. The ACMG lists 28 medically actionable cancer related genes, which are recommended to be reported to patients unrelated to the indication in clinical genetic testing. Does your institution report secondary findings in additional cancer related genes, which are not listed on the ACMG list? (e.g. *ATM, CHEK2*,...)
   1. My institution has an “in-house” gene list. Please add, if other (additional) cancer related genes are included in the "in house list" (e.g. *ATM, CHEK2*,...) or list genes, that are not included in the "in house list".
   2. My country has a national guideline or agreement for the reporting of secondary findings, which includes additional cancer related genes. Please add additional cancer related genes in which your institution looks for and reports secondary findings.
6. What are your institutions main reasons, for reporting secondary findings? If you report them due to guidelines, please provide the name of the guideline used.
7. What are your institutions main concerns when reporting secondary findings?
8. This question only refers to cancer related genes. How often do you encounter secondary findings in ACMG cancer related genes when performing genetic testing?
9. This question only refers to cancer related genes. Which variant classes are reported as secondary findings in cancer related genes in your institution?
   1. Your institution also reports variants of unknown significance (C3) as secondary findings in cancer releated genes. Please provide a short explanation why variants of unknown significance (C3) are reported as secondary findings in your institution.
   2. Your institution only reports pathogenic variants (C5) as secondary findings in cancer related genes. Please provide a short explanation why only pathogenic variants (C5) are reported as secondary findings in your institution.
10. Do you look for and report secondary findings in the 81 ACMG genes when performing trio-exomes/trio-genomes? Trio exome/genome sequencing analyses the sequences of three individuals: an index-patient and samples of two first-degree relatives, usually the parents. The goal is to differentiate de novo from inherited variants, and provide the segregation pattern in the family. This makes finding variants that explain the index patients genetic condition more efficient but can lead to secondary findings both in the index patient and the healthy relatives.
    1. This question refers to trio exomes/genomes: How are secondary findings examined in trio exomes/genomes in your institution?
11. Does the informed consent form in your institution work with an opt-in or an opt-out clause for the reporting of secondary findings?
12. Is specific information concerning secondary findings (e.g. the classes of conditions, possible benefits and disadvantages, general medical implications) provided to patients before genetic testing?
    1. If specific information concerning secondary findings is always provided to patients prior to genetic testing - who provides the specific information?
    2. If no or only limited specific information about secondary findings is provided prior to testing, please specify why:
13. Is specific genetic counselling (e.g. on the specific condition, medical consequences, psychosocial impact, and family implications) offered to patients after a secondary finding is reported?
    1. Is specific genetic counselling always offered to the patient after a secondary finding was reported, please specify who does the counselling
    2. When specific genetic counselling is provided after a secondary was reported - at what time point is it provided?
    3. If no or only limited genetic counselling for secondary findings is offered after a secondary finding was reported, please specify why:
14. Do you want to add some information or comment?
