# Supplementary material for: Reporting practices for secondary findings among ERN GENTURIS member institutions in 15 European countries
Source: Eur J Hum Genet. 2026 Mar 3;34(8):1121–9. doi: 10.1038/s41431-026-02044-7 (PMC13424559; doi:10.1038/s41431-026-02044-7)
Supplement: Supplementary file 2 — Supplement 2 [file 41431_2026_2044_MOESM2_ESM.docx]

Details on genes listed in “In-house” gene lists for the reporting of secondary findings. Some gene lists do also include genes listed on the ACMG v3.2 list.

| *ATM, CHEK2, RAD51C, RAD51D, BRIP1, BARD1, CDH1, CDKN2A, CDK4, BAP1* |
| --- |
| *ATM, CHEK2, BRIP1, BARD1, EPCAM, BLM* (homozygous cases), *CDKN2A, CDH1, NF1* |
| *APC, ATM, BAP1, BARD1, BLM, BMPR1A, BRCA1, BRCA2, BRIP1, CDH1, CDK4, CDKN2A, CHEK2, CYLD, DICER1, EPCAM, ERCC2, ERCC3, ERCC4, ERCC5, ERCC6, EXO1, FANCA, FANCB, FANCC, FANCD2, FANCE, FANCF, FANCG, FANCI, FANCL, FANCM, FH, FLCN, GRB7, KIT, MCPH1, MEN1, MET, MLH1, MLH3, MRE11A, MSH2, MSH6, MUTYH, NBN, NF1, NF2, NTHL1, PALB2, PMS2, POLD1, POLE, PRKAR1A, PTCH1, PTEN, RAD50, RAD51, RAD51C, RAD51D, RB1, RECQL, RECQL4, RET, SBDS, SDHAF2, SDHB, SDHC, SDHD, SLX4, SMAD4, SMARCA4, SMARCB1, STK11, SUFU, TMEM127, TP53, TSC1, TSC2, VHL, WRN, WT1* |
| *ABRAXAS1, ACD, ACTA2, ACTC1, ACVRL1, AIP, AKT1, ALK, ANKRD26, APC, APOB, ARID1A, ARID1B, ATM, ATP7B, ATR, ATRX, AXIN2, BAP1, BARD1, BLM, BMPR1A, BRAF, BRCA1, BRCA2, BRIP1, BTD, BUB1B, CACNA1S, CASQ2, CASR, CBL, CDC73, CDH1, CDK4, CDKN1B, CDKN1C, CDKN2A, CDKN2B, CEBPA, CHEK2, COL3A1, COL7A1, CREBBP, CSF3R, CTC1, CTNNA1, CTNNB1, CTR9, CTRC, CYLD, DDB2, DDX41, DICER1, DIS3L2, DKC1, DNAJC21, DNMT3A, DNMT3B, DOCK8, DSC2, DSG2, DSP, EGFR, ELANE, ENG, EPCAM, ERBB2, ERCC1, ERCC2, ERCC3, ERCC4, ERCC5, ERCC6L2, ETV6, EXT1, EXT2, EZH2, FANCA, FANCB, FANCC, FANCD2, FANCE, FANCF, FANCG, FANCI, FANCL, FANCM, FAS, FAT4, FBN1, FH, FLCN, FLNC, FOCAD, FOXE1, GAA, GALNT12, GATA1, GATA2, GBA, GJB2, GLA, GNA11, GNAQ, GPC3, GREM1, H19, HAVCR2, HAX1, HFE, HMBS, HNF1A, HOXB13, HPS1, HRAS, IDH1, IDH2, IKZF1, IPMK, ITK, JAK2, KCNH2, KCNQ1, KCNQ1OT1, KIF1B, KIT, KMT2C, KMT2D, KRAS, LDLR, LIG4, LMNA, LZTR1, MAD2L2, MAP2K1, MAP2K2, MAP3K1, MAX, MDH2, MEN1, MET, MITF, MLH1, MLH3, MMS19, MRE11, MSH2, MSH3, MSH6, MTAP, MTOR, MUTYH, MYBPC3, MYH11, MYH7, MYL2, MYL3, NBN, NF1, NF2, NHP2, NOP10, NRAS, NSD1, NTHL1, OTC, PALB2, PARN, PAX5, PCSK9, PDGFRA, PDGFRB, PHOX2B, PIK3C2G, PIK3CA, PIK3R1, PIK3R2, PKP2, PMS2, POLD1, POLE, POLH, POT1, PPP1CB, PRF1, PRKAG2, PRKAR1A, PRKN, PRSS1, PTCH1, PTEN, PTPN11, RAD50, RAD51, RAD51B, RAD51C, RAD51D, RAF1, RB1, RECQL, RECQL4, RELN, REST, RET, RHBDF2, RINT1, RIT1, RMRP, RNF43, RPE65, RPL11, RPL15, RPL23, RPL26, RPL27, RPL31, RPL35A, RPL36, RPL5, RPS10, RPS15, RPS17, RPS19, RPS20, RPS24, RPS26, RPS27, RPS27A, RPS28, RPS29, RPS7, RTEL1, RUNX1, RYR1, RYR2, SAMD9L, SBDS, SCN5A, SDHA, SDHAF2, SDHB, SDHC, SDHD, SEC23B, SETBP1, SH2B3, SH2D1A, SHOC2, SLC5A5, SLX4, SMAD3, SMAD4, SMARCA4, SMARCB1, SMARCE1, SOS1, SOS2, SPINK1, SPRED1, SQSTM1, SRGAP1, STAT1, STAT3, STK11, STN1, STX11, STXBP2, SUFU, TBXT, TERC, TERF2IP, TERT, TGFBR1, TGFBR2, TINF2, TMEM127, TMEM43, TNFRSF11A, TNIP1, TNNI3, TNNT2, TP53, TPM1, TRDN, TRIM28, TRIM37, TRIP13, TSC1, TSC2, TSR2, TTN, UBE2T, UNC13D, VHL, WAS, WRAP53, WRN, WT1, XPA, XPC, XRCC2, YAP1* |
| *CHEK2, ATM, BARD1, CDH1, BRIP1, RAD51C, RAD51D* and in single cases others. |
| *ATM, BARD1, BRIP1, CDH1, CDKN2A, CHEK2, RAD51C, RAD51D*, SDHA |
| *ATM, CHEK2, RAD51C, RAD51D, TINF2, PTCH1, SUFU, CTNNA1, ACD, AXIN2, BAP1, BARD1, BRIP1, CDH1, CDK4, TERTPOLD1, POLE, POT1, MET, CDKN2A, DICER1, EPCAM* (deletions)*, FH, FLCN, RNF43, RPS20, GREM1* (duplication) |
| *ATM, CHEK2* (only frameshifts*), RAD51B, RAD51C, NTHL1* |
| *ATM, BAP1, BARD1, BRIP1, CDH1, CDKN1B, CDKN2A, CHEK2, CTNNA1, DICER1, FH, FLCN, HOXB13, MET1, NF1, RAD51D, RAD51C, SDHA, SMARCA4, SMARCB1, SUFU, TERT* |
| *ATM, BARD1, CHEK2*, and *BRIP2*, 3' *EPCAM* del |
| *ATM, CHEK2, RAD51C, RAD51D* |
| *FLCN* |

Gene lists used as national guidelines or agreements for the reporting of secondary findings. Some gene lists do also include genes listed on the ACMG v3.2 list.

| *ATM, APC, BARD1, BRCA1, BRCA2, BRIP1, CDH1, CHEK2, EPCAM, MLH1, MSH2, MSH6,MUTYH, NBN,PALB2, PMS2, PTEN, RAD50, RAD51C, RAD51D, STK11, TP53* |
| --- |
| Version 3 (v3) of the comprehensive solid tumor and hematological tumor gene panel (STHTGP) contains the coding regions and 11 base pairs (bp) of flanking intronic sequence of 380 cancer-related genes. A complete gene list can be found on the BRIGHTcore website at: http://www.brightcore.be/gene-panels RefSeq with A of the ATG translation initiation codon as position +1.  The hereditary breast and ovarian cancer (HBOC) test reports the following: class 4 and 5 variants of the fully coding sequences and parts of the flanking introns (+/-11 bp) in the genes *BRCA1* (NM_007294.3), *BRCA2* (NM_000059.4), *CHEK2* (NM_007194.4), *PALB2* (NM_024675.3) and *TP53* (NM_000546.5). This core gene panel is supplemented with reporting of truncating and splice site changes in the genes *ATM* (NM_000051.3), *BRIP1* (NM_032043.2), *RAD51C* (NM_058216.2) and *RAD51D* (NM_002878.4); and class 4 and 5 variants in the *BARD1* (NM_000465.4), *MLH1* (NM_000249.3), *MSH2* (NM_000251.2) and *MSH6* (NM_000179.2) genes.  The germline hereditary colon cancer test reports the following: class 4 and 5 variants of the complete coding sequences and parts of the flanking introns (+/-11 bp) in the *APC* (NM_000038.6), *MLH1* (NM_000249.3), *MSH2* (NM_000251.2), *MSH6* (NM_000179.2), *MUTYH* (NM_001128425.2), *POLD1* (NM_002691.3), *POLE* (NM_006231.4), PTEN (NM_000314.8), *PMS2* (NM_000535. 7 with reporting always subject to reservations due to pseudogenes that interfere with reliable identification of *PMS2* variants) and *STK11* (NM_000455.4). |
| *BAP1, BARD1, BLM, BMPR1A, BRIP1, CDH1, CDK4, CDKN2A, CYLD, DICER1, DPYD, EGFR, EPCAM, FANCA,B,C, FANCM, FH, FLCN, CHEK2, KIT, MET, NBN, NF1, POLD1, POLE, SMARCA, WRN, SUFU, PTCH1, RAD51C, RAD51D, HOXB13* |
